# Mechanical Properties of Dual-Layer Electrospun Fiber Mats

**DOI:** 10.3390/polym17131777

**Published:** 2025-06-26

**Authors:** Ioana Caloian, Jocelyn Trapp, Bhalaji Yadav Kantepalle, Patrick Latimer, Timothy J. Lawton, Christina Tang

**Affiliations:** 1 Chemical and Life Science Engineering, Virginia Commonwealth University, Richmond, VA 23284, USAtrappj@vcu.edu (J.T.); latimerpa@vcu.edu (P.L.); 2 U.S. Army Combat Capabilities Development Command Soldier Center, Natick, MA 01760, USA

**Keywords:** layer, electrospinning, Young’s modulus, rule of mixtures, mechanical properties, polyurethane

## Abstract

Electrospinning with sequential layer deposition has been reported for various applications such as tissue scaffolds, shape memory materials, and separations. However, the effect of layering on the mechanical properties is not fully understood. In this work, layered structures of thermoplastic polyurethane (TPU) and nylon were selected as a model system to investigate the effect of sequential layer deposition on mechanical properties. Evidence of the layered structure was indicated by scanning electron microscopy (SEM) and Fourier Transform Infrared spectroscopy (FTIR) experiments. Layering TPU with nylon resulted in a 60-fold increase in the Young’s modulus. The Young’s modulus of the layered structure was reasonably predicted by the Voigt rule of mixtures. Furthermore, the Young’s modulus changes without any statistically significant change in elongation at break compared to a single layer of nylon. Thus, the elongation at break was dictated by the stiffer material, despite being present at a lower volume fraction. Overall, electrospinning with sequential layer deposition electrospinning is an effective approach for tuning the mechanical properties and surface chemistry of electrospun materials independently, which may be of interest for applications in tissue engineering and separations.

## 1. Introduction

Electrospinning is considered a straightforward, versatile approach to produce fibers with diameter on the order of 10–1000 nm [1,2,3]. Electrospinning utilizes electrostatic forces rather than mechanical forces used in conventional melt-spinning techniques to draw fibers [4]. In electrospinning, an electric field is applied between a polymer solution and collector to overcome surface tension [5]. Once surface tension is overcome, the polymer travels to the collector and the solvent evaporates, resulting in fiber formation. The fibers are deposited in a nonwoven mat [4]. The resulting nonwoven structures have high specific surface area and are of interest for applications in filtration, sensing, tissue engineering, drug delivery, etc. [1,2,3].

However, the weak mechanical properties of the electrospun material can limit practical applications [6,7,8]. Methods to control the fiber alignment and hierarchical structure of the fibers can be used to tune the mechanical properties of the fiber mat [3,7]. Additionally, the fiber composition, electrospinning parameters, and post-treatment (e.g., crosslinking) methods can be used to affect mechanical properties and have been reviewed elsewhere [6]. Enhancing the mechanical properties of electrospun fiber mats remains a significant challenge.

Dual-electrospinning of hybrid materials is considered a promising approach to improve the mechanical properties of an electrospun material compared with a single material [7,8,9,10]. Hybrid materials prepared with multi-nozzle techniques can enhance materials selection since materials from different solvents can be combined [9,11]. In dual-electrospinning, hybrid material mats can be prepared by using two spinnerets oriented perpendicular to a rotating drum collector. The orientation of the spinnerets can be controlled with a robotic setup [9,12]. Simultaneous spinning from both spinnerets results in two different polymeric fibers that are uniformly distributed and tightly interwoven/entangled with each other throughout the fiber mat [13]. Dual-electrospun mats have been used for applications in tissue engineering [11], wound healing [10,14,15], controlled drug release from implants [16], shape memory materials [17,18], and biodegradable nonwovens [19].

This dual-electrospinning approach has been useful for tuning the mechanical properties of the fiber mat [7,8,9,10]. For example, dual-jet electrospinning of poly(carbonate urethane) and poly(D,L-lactide-co-glycolide) has been performed to achieve properties that were a combination of both components [19]. The mechanical properties depended primarily on the thermoplastic component to the yield point; high values of elongation at break were observed due to the elastic component [19]. Similar experiments have been performed with other combinations of polymers such as polystyrene and nylon [20] as well as nylon and polyurethane [9]. When polycaprolactone was combined with polylactic acid, an increase in Young’s modulus was observed [8]. Addition of gelatin to chitosan/hyaluronic acid fiber systems was found to increase the ultimate tensile strength of the fiber mats [10]. Simultaneously electrospinning two solutions can require a large footprint and complex robotic system [9]. Notably, since the fibers are interwoven, this technique results in changes in mechanical properties and apparent surface chemistry [10].

An alternative approach to combine two materials has been sequential layer deposition (sometimes called sequential layer-by-layer electrospinning). Hybrid materials with layered structures, e.g., two-layer structures [21], sandwich structures [18,22], and multilayer structures [23,24], have been prepared by sequential layer deposition for various applications such as tissue scaffolds [23,24], lithium ion batteries [22], air filtration [25], water desalination [26], oil–water separation [21], and shape memory materials [18]. However, methods such as crosslinking [21] or introducing inorganic particles [23] have been used enhance mechanical properties. Thus, the effect of the sequential layer deposition on the mechanical properties of the resulting layered structure is not fully understood.

In this work, the use of electrospinning with sequential layer deposition to tune the mechanical properties and surface chemistry of electrospun materials independently was investigated. Thermoplastic polyurethane and nylon were selected as a model system based on their mechanical properties and ability to form fibers by electrospinning. The goals of this work were to prepare layered structures of polyurethane and nylon using sequential layer deposition electrospinning and use Attenuated Total Reflection–Fourier Transform Infrared (ATR-FTIR) spectroscopy to investigate the surface chemistry. The mechanical properties (e.g., Young’s modulus) were evaluated using tensile testing. To evaluate the effect of sequential layer deposition, the mechanical properties were compared to a sample of individually prepared polyurethane and nylon that were manually layered. For applications, the rule of mixtures was investigated as an approach to predict the stiffness of the layered structures.

## 2. Materials and Methods

As a model system, we selected one stiff polymer and one flexible polymer. Both polymers were commercially available and known to form uniform fibers by electrospinning so that the focus of this work was the effect of sequential layer deposition on changes in mechanical properties. Changes in stiffness as measured by the Young’s modulus of the layered structure were of particular interest. As a stiff polymer, nylon 6,6 was selected. Nylon 6,6 has many commercial applications including construction materials and fibers due to its high stiffness and high strength [27]. Electrospinning nylon fibers has been widely reported, e.g., [28,29,30]. As a flexible polymer, polyurethane was selected. Polyurethane is a copolymer composed of soft segments and hard segments connected by a urethane linkage. The soft segments impart flexibility; the hard segments provide strength. Thermoplastic polyurethanes (TPUs) typically include 60–85% soft segments [4,31]. Commercially available TPU from Covestro (Texin^®^ RxT70A, Covestro (Pittsburgh, PA, USA)) was selected since it is considered flexible [32]. Other grades of Texin have been electrospun [33]. The fiber size that can be achieved using solvent-based processing is smaller than melt processing [4,34]. Thus, nylon and TPU were selected for electrospinning with sequential layer deposition.

Nylon 6,6 pellets (Sigma-Aldrich 429171, relative viscosity 200–300), formic acid (reagent grade), and zinc chloride were obtained from Sigma-Aldrich (St. Louis, MO, USA). HPLC-grade tetrahydrofuran (THF) was obtained from Fisher Scientific (Pittsburg, PA, USA). Aromatic polyether-based thermoplastic polyurethane (TPU) pellets (Texin^®^ RxT70A, melt flow index 32 g/10 min) were obtained from Covestro (Pittsburgh, PA, USA). All materials were used as received.

For electrospinning, a nylon solution (14 wt.%) was prepared in formic acid by stirring at room temperature until macroscopically homogenous. Zinc chloride (0.1 wt.%) was dissolved in THF at ambient conditions. TPU (10 wt.%) was dissolved in the prepared zinc chloride/THF solution by stirring at room temperature until macroscopically homogenous. Typical electrospinning parameters for TPU were a flow rate of 1 mL/h, tip-to-collector distance of 10 cm, and a voltage of 14 kV using a 22-gauge blunt tip (0.508 mm i.d.). Typical electrospinning parameters for nylon were a flow rate of 0.1 mL/h, tip-to-collector distance of 7 cm, and an operating voltage of 17 kV using a 22-gauge blunt tip (0.508 mm i.d.).

A custom-designed electrospinning setup was used as previously described [3,35]. Briefly, the setup consisted of a high-voltage power supply (Matsusada Precision Inc., model AU-40R0.75 with positive polarity, Kusatsu, Shiga, Japan), a syringe pump (New Era NE-300: Farmingdale, NY, USA), and an aluminum plate collector (11″ diameter). The plate was mounted on a custom-built acrylic base. The syringe was horizontally fixed on the syringe pump and mounted so that the tip of the needle was level with the center of the plate. The plate was covered with aluminum foil with a non-stick coating (Reynolds Consumer Products, Lake Forest, IL, USA). The setup was housed in a fume hood and operated under ambient conditions (i.e., temperatures between 19 and 22 °C and 26–47% relative humidity).

Layered structures of TPU and nylon were prepared using a sequential layer deposition approach. The first polymer solution was electrospun and deposited on the plate. Subsequently, the second polymer solution was electrospun. The position of the plate was manually adjusted so that the resulting fibers were collected on top of the first layer. TPU was collected as the first layer to ease removal from the foil; nylon was collected as the second layer. Each layer was collected for 2 h.

The layered structures samples spun using the sequential layer deposition approach were compared to a layer of nylon as well as a layer of TPU electrospun separately and layered manually. Specifically, nylon (14 wt.%) was electrospun for 2 h and collected on aluminum foil with a non-stick coating. Separately, TPU (10 wt.% in 0.1 wt.% ZnCl in THF) was electrospun for 2 h and collected on aluminum foil with a non-stick coating. Following electrospinning, samples of each material were cut into 10 mm × 80 mm rectangles and removed from the foil. A rectangle of TPU was layered with a rectangle of nylon and manually pressed together (finger strength).

The electrospun samples were characterized with scanning electron microscopy (SEM, Hitachi SU-70 FE-SEM (Tokyo, Japan)). For analysis, the samples were sputter-coated with platinum, then imaged using an accelerating voltage of 5 kV. The average fiber size and standard deviation were determined by measuring the diameter of 100 fibers using ImageJ software version 1.53 (US NIH). The top and bottom of the multilayer samples were analyzed by Attenuated Total Reflection (ATR) infrared spectroscopy (Thermo Scientific Nicolet iS10, Thermo Fisher Scientific, Waltham, MA, USA). Spectra were recorded from 400 to 4000 cm^–1^ averaging 32 scans.

Tensile tests were performed on rectangular-shaped samples (10 mm × 80 mm) using a Universal Testing System (Intron model 3343, 100 N load cell, Instron Corporation, Canton, MA, USA). The sample thickness was measured with calipers. The thickness of the specimen was the average thickness measured at three different points. Based on previous reports, samples were mounted between paper frames [36]. Briefly, paper frames with outer dimensions of 20 mm × 80 mm and a 12.7 mm × 12.7 mm area removed from the center were prepared. Both ends of the specimen were adhered to the paper frames using double-sided, thin Scotch tape (3M Scotch Magic Tape (Matte Finish) 3/4”x36 yards Desk Dispenser Refills). After attaching the paper frame and sample to the tensile-testing machine, the sides of the paper frame were cut. The tests were performed at a crosshead speed of 0.381 mm/min until fracture occurred or a maximum collection time of 2 h was reached. Representative nominal stress–strain curves were reported for each sample. The Young’s modulus was calculated from the initial part of the slope from engineering stress–strain curves [36]. A minimum of 4 samples of each material were analyzed and the average values of the mechanical parameters and standard deviations were reported.

## 3. Results and Discussion

**Figure 1:**
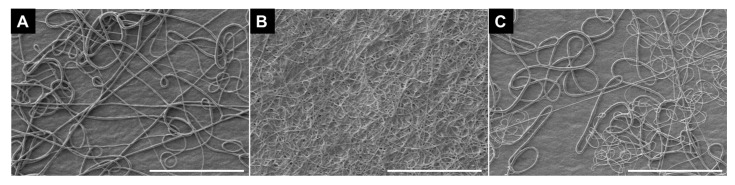
SEM micrographs of (**A**) a single layer of electrospun TPU (10 wt.% in THF with ZnCl), (**B**) single layer of electrospun nylon (14 wt.% in formic acid), and (**C**) layered structure of TPU and nylon. Scale bars represent 100 microns.

Since electrospinning of polyurethane and nylon has been well established (e.g., [30,31]), solutions for electrospinning (fibers free from bead defects) were formulated based on the literature. Electrospinning TPU in THF was attempted from the solubility limit (15 wt.%) to 1 wt.% based on previous reports [37,38]. Beaded fibers were observed at 5 wt.% and droplets were observed at 1 wt.% (Supporting Information, Figure S1A,B). Fibers were obtained at 10 wt.% (Supporting Information, Figure S1C). A transition from beads to beaded fibers to fibers with increasing concentrations is consistent with the onset of polymer entanglement, which has been correlated to the formation of fibers via electrospinning [30,39]. However, significant needle clogging was observed that interfered with continuous fiber collection. The needle clogging has been attributed to high solvent volatility and low solvent dielectric constant/solution conductivity [37]. To increase the solution conductivity, a salt, zinc chloride, was introduced into THF, since salts are commonly used electrospinning additives known to increase the conductivity of polymer solutions [40]. The addition of the zinc chloride reduced needle clogging and continuous collection of fibers was achieved. SEM of the resulting TPU fibers spun from THF with zinc chloride are shown in Figure 1A. This result was attributed to the increased net charge density of the solution with the addition of salt and is consistent with previous reports of electrospinning polyurethane from solvent mixtures [37].

Nylon nanofibers were prepared by electrospinning a solution of nylon in formic acid. Formic acid is a commonly used solvent for electrospinning nylon [41,42]. As a guide for the formulation of electrospinning solutions, the entanglement concentration has been estimated to be between 10 and 15 wt.%. Thus, in a typical experiment, nylon 6,6 was dissolved in formic acid (14 wt. %). Electrospinning of 14 wt.% nylon in formic acid resulted in uniform fibers generally free from bead defects (SEM of the resulting nylon fibers spun from formic acid are shown in 1B) (high-magnification image, Figure S2). This concentration is consistent with ranges previously reported for electrospinning [41,42].

Fibers without bead defects were obtained for both TPU and nylon. The nylon fibers were smaller than the TPU fibers. The average fiber diameter for nylon fibers were 0.32 ± 0.23 µm, whereas the average fiber diameter for the TPU fibers was 1.72 ± 0.90 µm.

**Figure 2:**
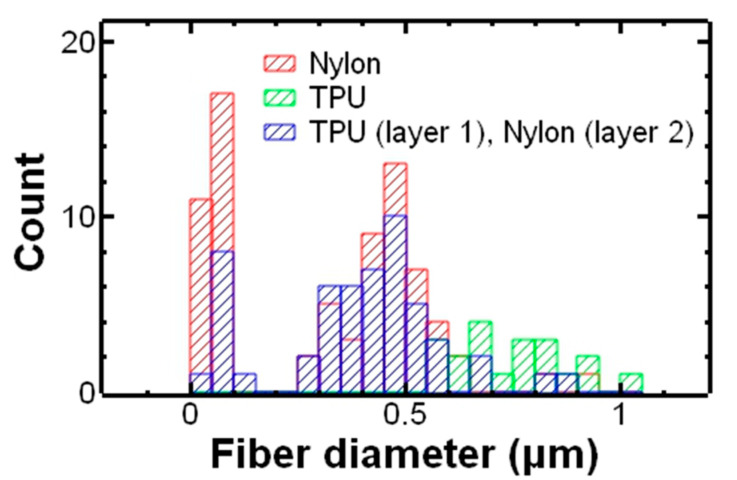
Fiber size distribution of nylon (electrospun in a single layer), TPU (electrospun in a single layer), and a layered structure of TPU and nylon.

Layered structures were prepared by first depositing an electrospun layer of TPU, followed by electrospinning a layer of nylon atop the TPU layer. Electrospinning nylon onto TPU did not significantly affect the electrospinning process and the parameters, e.g., voltage, flow rate, were comparable to electrospinning nylon as a single layer. SEM of the layered structure is shown in Figure 1C. Minimal bead defects were observed. Notably, the fiber size appears bimodal (small and large fibers). The fiber size distribution of the electrospun layered structure sample is compared to the single layer of TPU and nylon in Figure 2. The fiber size distribution of the layered structure (blue bars) indicates the presence of nylon fibers (red bars) as well as TPU fibers (green bars). Overall, the layered structure sample had an average fiber diameter of 0.78 ± 0.66 µm. The average fiber size of the layered structure falls between TPU and nylon, as expected, and is statistically significant based on a Student’s t-test (*p* < 0.05). This result suggested that the fibers observed were likely a combination of nylon as well as TPU fibers.

**Figure 3:**
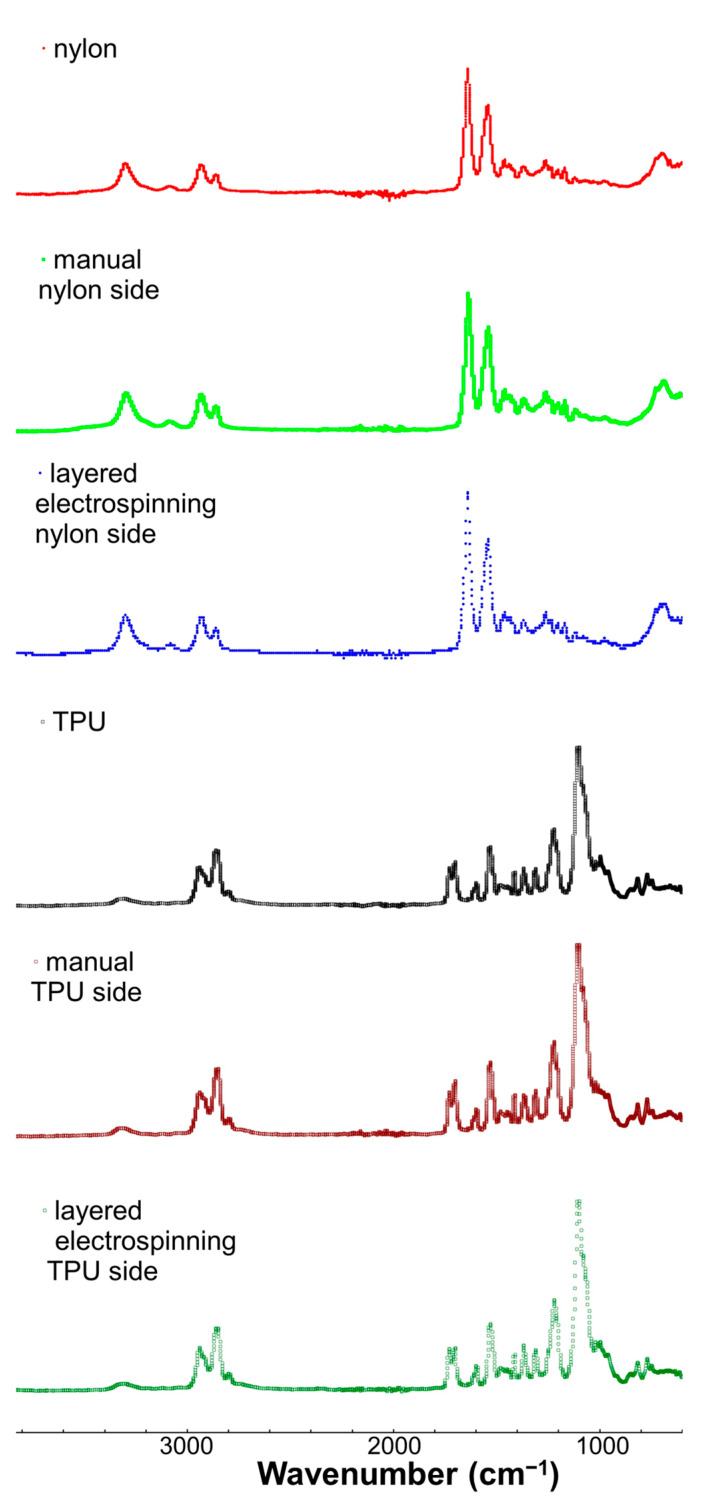
ATR-FTIR spectra of nylon (single layer), nylon surface of a layered structure prepared by manual layering, nylon surface of a layered structure prepared by sequential layer deposition electrospinning, TPU (single layer), TPU surface of a layered structure prepared by manual layering, and TPU surface of a layered structure prepared by sequential layer deposition electrospinning. All spectra are baseline-corrected and offset for clarity.

To examine the surface chemistry of the layered structures, ATR-FTIR was performed. Specifically, the two surfaces of the electrospun layered structures of TPU:nylon were analyzed by ATR-FTIR spectroscopy (surface analysis). The layered structures prepared by sequential layer deposition electrospinning were compared to separately prepared layers that were manually layered. For comparison, the spectra of single layers of electrospun TPU and nylon were also analyzed. The IR spectra of the samples (offset for clarity) are shown in Figure 3.

The IR spectrum of the TPU surface of the TPU:nylon-layered structure (bottom, dark green) showed absorption peaks associated with the amide bond in TPU at 1700 and 1200 cm^−1^. Multiple peaks have been attributed to the stretching vibration of C=O in the amide bond, the bending vibration of N-H in the amide bond, and the C–N’s stretching vibration of the amide bond. These peaks occur in the same region as the C=C skeleton vibration of aromatic benzene ring at 1600 cm^−1^, as well as the stretching vibration of the C=O bond from the ester group at 1730 cm^−1^ and the stretching vibration of C–O–C at 1100 cm^−1^. Due to vibration coupling, multiple peaks around 1100 cm^−1^ were observed. Additional peaks around 2800–2900 cm^−1^ have been attributed to the stretching vibrations of CH_2_ [38]. The peak between 3400 and 3500 cm^−1^ has been attributed stretching of -OH due the presence of moisture on the surface [43].

The IR spectrum of the nylon surface of the TPU:nylon-layered structure (blue spectrum) showed absorption peaks characteristic of polyamide fibers. Specifically, peaks around 3300 cm^−1^ due to N-H stretching of the amorphous phase were observed [44]. Amide I and amide II peaks between 1650 cm^−1^ and 1500 cm^−1^ were also observed. The intensity of the amide I peak was greater than the amide II, which is consistent with previous observations [44]. Amide-III at 1370 cm^−1^, O=C–H at 581 cm^−1^, and C–C at 687 cm^−1^ were also observed. The broad peak between 3400 and 3500 cm^−1^ can be attributed to a stretching of -OH and to physiosorbed moisture on the surface [44].

Overall, the IR spectra of each side of the layered structure were consistent with the single-layer samples of the same material and manually layered samples. These results indicated that the chemical structure of the TPU and nylon layers (at the surfaces away from the interface) were not significantly affected by sequential layer deposition electrospinning; no evidence of chemical reactions between TPU, nylon, or the solvents was observed. This result is important because it indicates that layered structures can be achieved and maintain comparable surface chemistry to the individual fiber layers.

**Figure 4:**
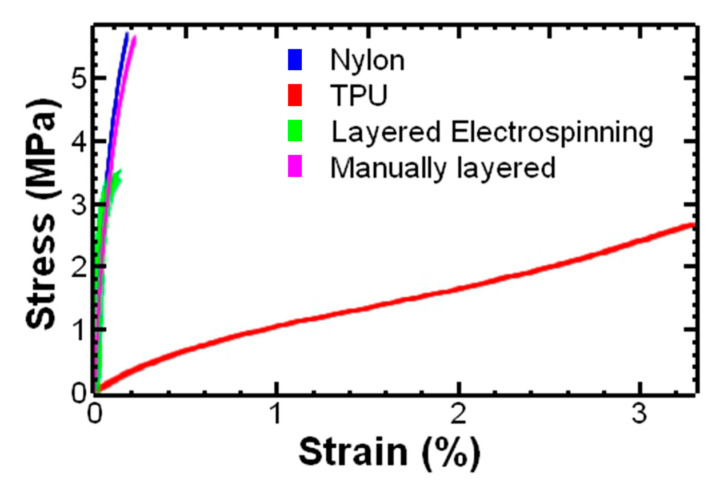
Representative engineering stress–strain curves of electrospun TPU and nylon compared to TPU–nylon-layered structure prepared by electrospinning of sequential layer deposition and TPU–nylon-layered samples prepared by manually layering individually prepared layers.

Representative engineering stress–strain curves for electrospun nylon and TPU were compared with TPU–nylon-layered structures prepared by electrospinning with sequential layer deposition as well as manually layered samples (Figure 4). The stress–strain behavior of nylon showed an initial linear behavior followed by a yield response [45]. The stress–strain behavior of the TPU was expected for a thermoplastic elastomer (Figure 4, red curve). A linear region was observed below 15% strain and strain hardening was observed above 250% strain. Strain hardening has been commonly observed upon the uniaxial tension of thermoplastic polyurethane [46]. Polyurethane is a copolymer of hard segment-enriched domains dispersed in a matrix of soft segments [31]. Strain hardening has been attributed to breakage and rearrangement of the hard domains into more numerous domains effectively increasing the density of physical crosslinks, resulting in an increase in stiffness [47]. The stress–strain behavior was consistent with previous reports of TPU films [48].

The response of the TPU–nylon-layered structures prepared manually (Figure 4, pink curve) appeared similar to the nylon (Figure 4, blue curve). An initial linear behavior was observed followed by a yield response; the ultimate strength may be affected by both the nylon and TPU components in the sample. The response of the layered structure sample prepared by electrospinning with sequential layer deposition (Figure 4, green curve) also appears similar to the nylon with an initial linear behavior followed by a yield response; the TPU layer had minimal effect.

The Young’s modulus, ultimate stress, and elongation at break of the samples were determined from the stress–strain curves. The TPU samples did not yield before the end of the test. Thus, the elongation at break for TPU was determined to be >310% (Table 1). This result is consistent with previous reports of polyurethanes [9,49]. The flexible segments of the thermoplastic polyurethanes (TPUs) provide elastomeric properties such as high elongation at break [50]. The Young’s modulus of the TPU fiber mats was 1.2 ± 0.2 MPa (Table 1). This result was consistent with previous reports of electrospun TPU mats using commercially available polyurethanes [49]. The stiffness was also comparable to Texin^®^ RxT70A (Covestro, Pittsburgh, PA, USA) films prepared by solvent casting [51].

In contrast, the Young’s modulus of the nylon fiber mats was 202.6 ± 95.3 MPa, which was significantly higher than the TPU fiber mats (*p* < 0.0001) with much lower elongation at break 24 ± 7%. The Young’s modulus and elongation at break were comparable to previous reports of electrospun nylon 6,6 [52].

**Figure 5:**
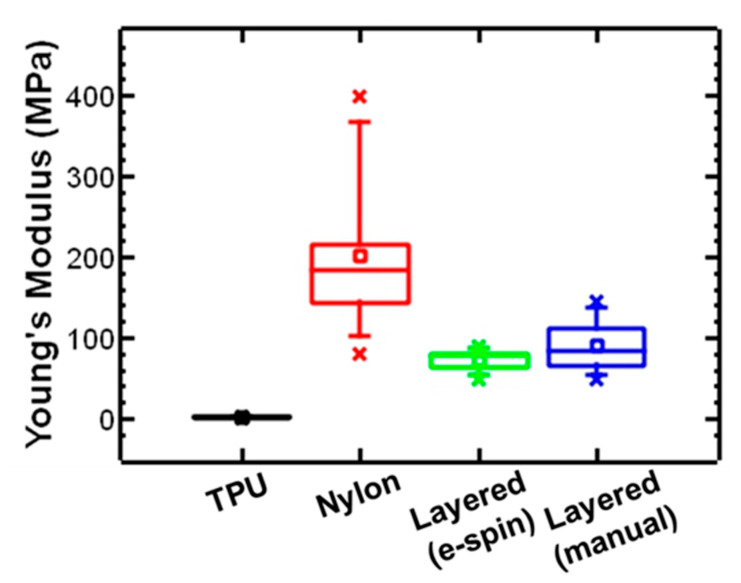
Box and whisker plot comparing Young’s modulus of electrospun TPU and nylon with layered structure TPU–nylon samples prepared by electrospinning or manually layering. The box represent lower to upper quartile, the line represents 5th% to 95th%, and the x symbol represents the maximum and minimum of each set of measurements.

The effect of sequential layer deposition on stiffness of the layered structure was investigated. The layered structure fabricated by sequential deposition of a layer of TPU followed by a layer of nylon showed an increase in Young’s modulus compared to TPU. The Young’s modulus increased 60-fold from 1.2 ± 0.2 MPa to 73.1± 12.5 MPa (*p* < 0.0001) (Figure 5). As expected, this value falls between the Young’s modulus for TPU and nylon.

To evaluate the effect of the sequential layer deposition, the mechanical properties of the layered structure were compared to an electrospun TPU layer as well as an electrospun nylon layer prepared separately and manually layered together. The modulus of the layered structure was slightly lower than the layered structure prepared by separately prepared layers manually pressed together (*p* = 0.12). These results suggest that deposition during electrospinning the second layer does not significantly affect the mechanical properties of the layered structure for the polymers/solvents used. In this case, an alternative approach may be to prepare layers separately before fabricating the layered structures with independently tunable mechanical properties and surface chemistry.

For the design of layered structures for various applications, it would be helpful to predict the stiffness (Young’s modulus). One approach to predicting the effective mechanical properties of a material made of multiple components would be to apply the rule of mixtures. The rule of mixtures is a simple approach that assumes all components experience identical, linear elastic deformation with an equivalent multilayer structure [53,54]. The properties (Young’s modulus) of the layered structure can be predicted based on the elastic modulus of each component and their respective volume fractions [53,55]. Specifically, for estimating the Young’s modulus (*E_overall_*) based on the Young’s modulus (*E_i_*) and volume fraction (*v_i_*) of each component [56], the Voigt rule of mixtures can be used assuming that each component undergoes the same strain as

(1) $$E_{overall}=\sum_{i}v_{i}E_{i}$$

The applicability of the rule of mixtures to the layered structures prepared in this work was investigated. Since no interaction between the polymers was indicated in ATR-FITR, only the properties of the TPU and nylon was considered in this analysis. The volume fraction of each layer was estimated based on the thickness of individually spun TPU and individually spun nylon samples. Based on the rule of mixtures, the layered structure of TPU and nylon would have a Young’s modulus of 68 MPa (within 10% of the experimentally measured value). The results suggest that the stiffness of the layered structures (as measured by the Young’s modulus) is affected by the properties of the layers. Thus, the rule of mixtures provides a simple and effective approach for predicting the Young’s modulus of the layered structures.

Interestingly, comparable results for increasing the modulus of polyurethane with PCL have been reported using dual-electrospinning [17]. Based on the data reported for dual-electrospun PCL and polyurethane, the Voigt rule of mixtures worked best at fractions of PCL (>30%). These results suggest that, at a volume fraction of ~30%, the fusion points between fibers achieved with dual-electrospinning or sequential layer deposition contribute minimally to the mechanical properties of the hybrid material in agreement with previous results [18]. Using these approaches, materials selection and the amount of each material appear to be important factors in the mechanical properties of the resulting electrospun structure. Further works investigating extending the rule of mixtures to predict the properties of layered structures made from other materials or layers of additional thicknesses are of future interest.

While the Young’s modulus was influenced by the TPU component and the nylon component, the elongation at break as well as the ultimate strain were comparable to nylon. Interestingly, based on thickness, nylon was the lower-volume fraction component. This result indicates that the strength and elongation at break of the layered structure was dictated primarily by the stiffer component despite being present at a lower volume fraction, which, in this case, is nylon. Interestingly, layered electrospun structures appear to be a unique approach to tune the mechanical properties independent of surface chemistry. Further increases in strength could be achieved by fiber drawing [57] or post-processing, e.g., selective component annealing [8], by using a nonwoven substrate, e.g., PP [25], by crosslinking [21] or by incorporating inorganic fillers [23].

## 4. Conclusions

Using TPU and nylon as a model system, electrospinning with sequential layer deposition was used to tune the mechanical properties and surface chemistry of layered systems independently. The Young’s modulus of the layered structure was compared to the individual layers. Interestingly, the rule of mixtures was a simple and effective method to predict the modulus of the layered structure. Furthermore, the Young’s modulus changes without any statistically significant change in elongation at break compared to a single layer of nylon. The strength and elongation at break of the hybrid material were dictated primarily by the stiffer component, despite being present at a lower volume fraction (i.e., thinner layer). This approach may be especially useful for applications in which independent tunability of mechanical properties and surface chemistry are of interest, e.g., tissue engineering [23,24], separations [21,26].

## Figures and Tables

**Table 1:** Summary of mechanical properties of layered structures of TPU and nylon prepared by electrospinning with sequential layer deposition compared to nylon (single layer), TPU (single layer), and a layered structure of TPU and nylon prepared by electrospinning layers separately and manually layering.

	Young’s Modulus (MPa)	Ultimate Strength (MPa)	Elongation at Break (%)
TPU *	1.2 ± 0.2	n.d. ^†^	>310
Nyon **	202.6 ± 95.3	16 ± 10	24 ± 7
TPU–Nylon-layered structure (manual) **	90.4 ± 31.2	8 ± 4	27 ± 6
TPU–Nylon-layered structure—electrospinning with sequential layer deposition electrospinning **	73.1 ± 12.5	6 ± 1	30 ± 21

* *n* = 4, ** *n* = 10, ^†^ not determined.

## Data Availability

The original contributions presented in this study are included in the article. Further inquiries can be directed to the corresponding author.

## Supplementary Materials

The following supporting information can be downloaded at: https://www.mdpi.com/article/10.3390/polym17131777/s1, Figure S1: SEM micrographs of electrospun TPU in THF. Figure S2: SEM of electrospun nylon in formic acid at high magnification.
