# Supplementary material for: Mechanical Properties of Dual-Layer Electrospun Fiber Mats
Source: Polymers (Basel). 2025 Jun 26;17(13):1777. doi: 10.3390/polym17131777 (PMC12251699; doi:10.3390/polym17131777)
Supplement: Supplementary file 1 [file polymers-17-01777-s001.zip › polymers-3703647-supplementary.pdf]

Supporting Information

Mechanical Properties of Dual-Layer Electrospun Fiber Mats

Ioana Caloian [1], Jocelyn Trapp [1], Bhalaji Yadav Kantepalle [1], Patrick Latimer [1], Timothy J. Lawton [2], and Christina Tang [1,*]

1 Chemical and Life Science Engineering, Virginia Commonwealth University, Richmond, VA
2 U.S. Army Combat Capabilities Development Command Soldier Center, Natick, MA

* Correspondence: ctang2@vcu.edu

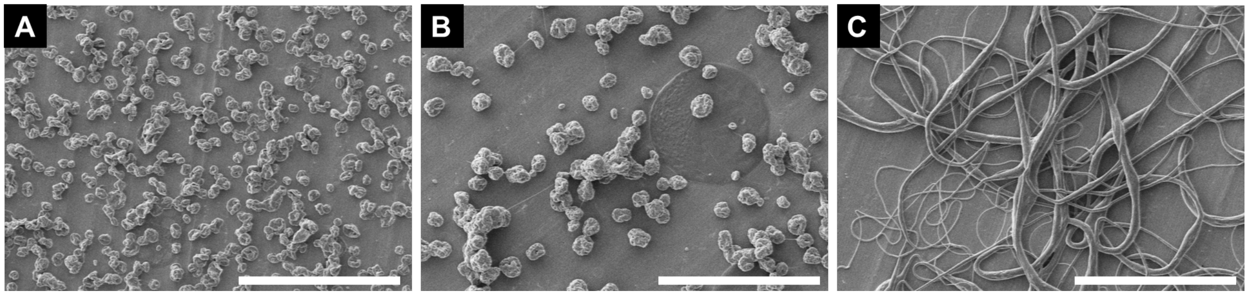

Figure S1. SEM micrographs of electrospun TPU in THF at (A) 1 wt.% (B) 5 wt.% (C) 10 wt.%. The transition from beads to beaded fibers to fibers with increasing concentration is the onset of polymer entanglement. Notably, for 10 wt.% needle clogging prevented continuous collection of fibers. Scale bars represent 100 microns.

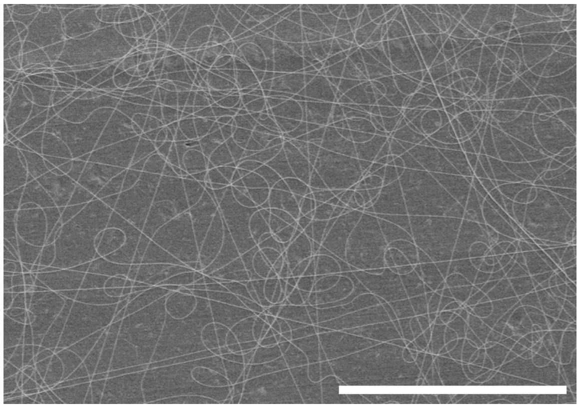

Figure S2. High magnification SEM micrograph of electrospun nylon (14 wt.% in formic acid). Scale bar represents 50 microns.
